# Environmental contamination by canine geohelminths

**DOI:** 10.1186/1756-3305-7-67

**Published:** 2014-02-13

**Authors:** Donato Traversa, Antonio Frangipane di Regalbono, Angela Di Cesare, Francesco La Torre, Jason Drake, Mario Pietrobelli

**Affiliations:** 1 Faculty of Veterinary Medicine, University of Teramo, Teramo, Italy; 2 Department MAPS, University of Padua, Padua, Italy; 3 Novartis Animal Health, Origgio, VA, Italy; 4 Novartis Animal Health, Greensboro, NC, USA

**Keywords:** *Toxocara canis*, *Ancylostoma caninum*, *Trichuris vulpis*, Faeces, Dog, Urban areas

## Abstract

Intestinal nematodes affecting dogs, i.e. roundworms, hookworms and whipworms, have a relevant health-risk impact for animals and, for most of them, for human beings. Both dogs and humans are typically infected by ingesting infective stages, (i.e. larvated eggs or larvae) present in the environment. The existence of a high rate of soil and grass contamination with infective parasitic elements has been demonstrated worldwide in leisure, recreational, public and urban areas, i.e. parks, green areas, bicycle paths, city squares, playgrounds, sandpits, beaches. This review discusses the epidemiological and sanitary importance of faecal pollution with canine intestinal parasites in urban environments and the integrated approaches useful to minimize the risk of infection in different settings.

## Review

Soil-transmitted helminthoses affects more than 2 billion people worldwide [1]. Other than human-specific parasites, intestinal nematodes affecting dogs have a relevant health-risk impact for both animals and human beings. The importance of these pathogens is often minimized by veterinarians and the general public, although *Toxocara canis*, hookworms (i.e. *Ancylostoma* spp.) and whipworms (i.e. *Trichuris vulpis*) are the most relevant canine helminths in terms of geographic distribution and clinical importance [2,3].

The presence of infective eggs or larvae in the environment has a crucial role among the different routes of transmission of dog intestinal nematodes in both humans and animals. In fact, human beings become infected by canine *Toxocara* spp. and *Ancylostoma* spp. most frequently via contaminated soil [4-7].

Studies from various countries have demonstrated a high rate of soil and grass contamination with infective parasitic elements in leisure, recreational, public and urban areas, i.e. parks, green areas, bicycle paths, city squares, playgrounds, sandpits, beaches.

When using these areas, people often take their pets with them. Owned dogs and stray animals may defecate in public streets and areas, thus contaminating the environment with parasites and favoring zoonotic transmission and (re-) infection for other animals.

While readers interested in biology, pathology and general control of canine intestinal nematodes are referred to [2,3,7-9], the present article reviews the epidemiological importance of faecal pollution in urban environments with canine intestinal parasites in terms of veterinary and human health and discusses the integrated approaches useful to minimize the risk of infection.

### The environment is incessantly contaminated

*Toxocara canis* and *Ancylostoma caninum* are, respectively, the primary species of roundworms and hookworms infecting dogs worldwide. Other species of ascarids and ancylostomatids may be present in particular areas, e.g. *Toxascaris leonina* in Europe and USA, *Uncinaria stenocephala* in colder areas of temperate and subarctic regions, and *Ancylostoma braziliense* in the southern hemisphere. Additionally, the whipworm *T. vulpis* is the ubiquitous whipworm inhabiting the large intestine of dogs [2,3].

Parasitic burdens and egg output are higher in puppies but patent intestinal infections may occur in dogs of all ages and categories [10-19], even when under regular control programs [15,20]. Bitches are a relevant source of infection for other animals and environmental contamination because they often harbor somatic larvae, which mobilize during pregnancies and infect subsequent litters even when re-infections do not occur. Puppies become infected *in utero* and *via* the milk, but a proportion of mobilized larvae reach adulthood in the intestine of the dam and cause a patent infection with a long-lasting high egg shedding [21,22]. The patent infection in the bitch can be re-enforced when suckling puppies defecate immature ascarids, which are ingested by the dam and become adults in her intestine [23]. Altogether these biological features make nursing bitches and puppies a very important source of environmental contamination by *T. canis*.

**Figure 1:**
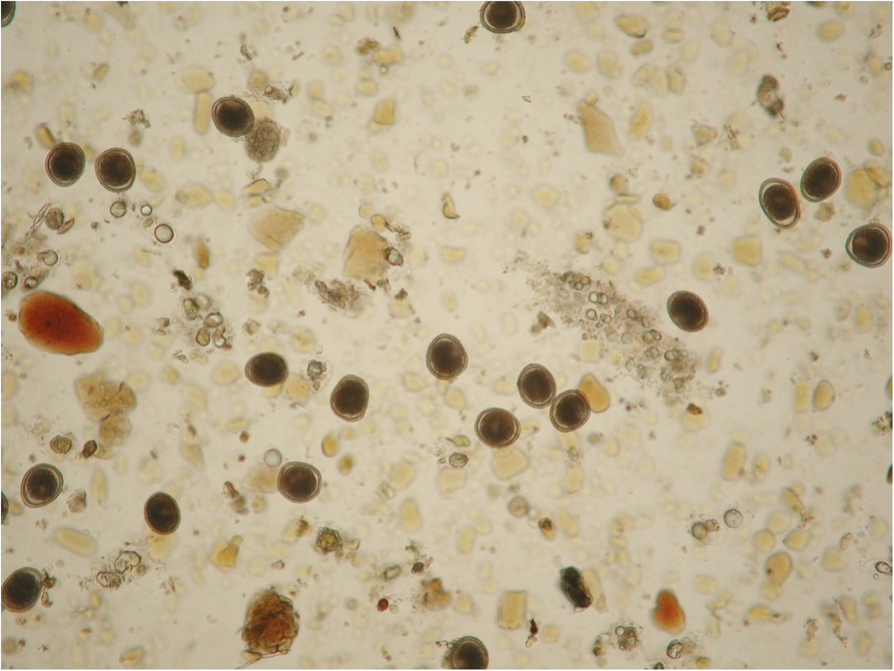
**Copromicroscopic examination of a puppy: microscopic field (10×) showing a high shedding of *Toxocara canis *** **eggs.**

Remarkably, pre-vaccination confinement of puppies would often imply that eggs are shed into the home or private gardens and backyards, thus posing a potential health risk for the owners [24]. This is of great importance considering that virtually 100% of puppies acquire toxocarosis by transmammary and/or transplacental route/s and that they pass thousands of *T. canis* eggs per gram of feces every day (Figure 1).

Hookworm filariform larvae present in the soil infect a suitable host by actively penetrating the skin (especially for *Ancylostoma* spp.) and/or *via* the oral route (i.e. *Ancylostoma* spp., *Uncinaria* spp.) [3,23,25-27]. As with *T. canis*, hypobiotic larvae may survive for years in the tissues of adult dogs and when reactivated during oestrus and in the last 2–3 weeks of pregnancy, they are passed *via* the milk to the litter [27-30]. Adult dogs may suffer patent ancylostomosis when they become infected with environmental larvae or when hypobiotic stages are re-activated by drivers of stress [3]. Remarkably, dogs infected by *A. caninum* may shed millions of hookworm eggs for weeks [7].

The absence of a vertical transmission in *T. vulpis*, its long pre-patent period and a partial ability to stimulate a protective immune response [31,32], explain the high degree of intestinal trichurosis in adult dogs rather than in puppies. Hence, it could be erroneously argued that this parasite is not spread as easily as roundworms and that the environment is not as contaminated by whipworm eggs.

It is estimated that the contamination of soil with *Toxocara* eggs may be more than the 90% of the investigated areas worldwide [33]. This is explained by the fact that mature eggs of ascarids (and *T. vulpis* as well) can survive in contaminated soil even in harsh conditions (e.g. they may resist to chemicals, broad temperature ranges and several degrees of moisture), thus are available for ingestion at any time by susceptible hosts [8,9,34]. Also, viability and infectivity of environmental larvated eggs persist for years, thus explaining the high number of chances that dogs have of becoming infected and the difficulties in controlling these intestinal parasitoses. As an example, eggs of *T. vulpis* survive from cold winter to hot summer, especially in wet and shady areas, which are widely distributed in green areas of metropolitan cities [9].

Larvated eggs of *T. canis* and larval ancylostomatids are an efficient environmental source of infection for various animals, which act as paratenic hosts. These animals greatly contribute to maintaining the biological cycle of toxocarosis and ancylostomosis everywhere. In fact, dogs can become infected by *Toxocara* by ingesting tissues of invertebrates (e.g. earthworms), ruminants (e.g. sheep), rodents, birds (e.g. chicken) [3,7,31].

The role of wildlife is another exogenous factor contributing to the environmental contamination. In fact, movements of wildlife to sub-urban and urban environments due to destruction or reduction of their habitat is another source of soil contamination by *T. canis*[35]. The key example is represented by synantropic fox populations, which reinforce environmental contamination and risk of infection for humans and stray and domestic dogs [36].

Thus, a combination of these factors is the basis for an extremely high environmental contamination and a life-long risk of infection for dogs living in contaminated areas.

The analysis of datasets from field investigations has recently described general principles and approaches useful to quantify levels of contamination with ascarid eggs and to prioritize control measures. In particular, the relative role of dogs, cats and foxes in disseminating parasite eggs in a given environment (i.e. the city of Bristol, UK) was investigated. This study, carried out in an urban setting in the absence of stray animals, showed that pet dogs are the source of most of the eggs that contaminate the environment [24]. Obviously, this study example would differ in terms of results and conclusions upon different localities, but in general it demonstrated that an estimation of egg density in urban settings is possible and provides local epidemiological models of egg outputs and sources of contamination. Also, this study illustrated that education of pet owners is crucial to minimize the risk of disease transmission to animals and humans and that stray dogs are not the culprits of faecal urban pollution in every city. It is obvious that the number of eggs contaminating the environment is dependent on the amount of faeces eliminated by owned and stray dogs and on the extent of feces removal by the owners. However, there is a lack of information on rates of deposition and removal of dog faeces from public spaces in several areas [24]. In this regard, recent field studies conducted during summer 2012 by operators observing dogs and their owners in parks and green public areas located in the cities of Rome and Padua (Italy), showed that 15.6% pet-owners did not remove dog faecal deposits from the ground, with a few differences between the investigated cities (13.5% and 16.9%, respectively) (unpublished data).

### Risk for humans

Human beings become infected by *T. canis* most commonly by ingesting embryonated eggs from the soil. Other sources of transmission with dog intestinal nematodes include ingestion of larvae resting in tissues of paratenic hosts, or hookworm larvae in contaminated soil, which can penetrate the skin of humans walking barefoot.

The presence of eggs on the ground is not only implicated with the direct infection for humans but could represent a source of contamination for pets’ coats. Indeed, the role of embryonated ascarid eggs present on the fur of dogs has been evocated as a source of human infections via hand-to-mouth contact [6,37,38].

Indeed, infective eggs have been found on the coat of dogs in different studies suggesting that direct contact with these animals could be a potential risk for humans. Eggs of *T. canis* may be present on the hair of both stray and privately owned dogs, with the latter considered as a more important risk for human infection due to the frequent contact with people [39]. On the other hand, close-contact with a pet has been considered an unlikely risk of infection with intestinal parasites for humans because the strong adherence of eggs on the animal’s fur, the relatively high number of eggs which should be ingested to establish an infection and the long time for the embryonation (i.e. minimum 2 weeks) [7,40]. Rather than a self-contamination (e.g. with self-grooming transmitting eggs from the peri-anal region to other parts of the body), dogs may pick up *Toxocara* eggs on their hair by the scent-rolling [6]. In any case, regarding the actual risk for human infection *via* touching or petting a pet, scent-rolling can be a relevant cause of contamination for the animals coat when a pet is taken out in contaminated areas. Interestingly, the presence of non-canine parasite eggs on the fur of dogs indicates that the contact with a contaminated environment plays a key role in the acquisition of eggs by the animals [41]. The presence of embryonated eggs on the fur of owned dogs in some studies [37,40,42,43] may account for a lack of care in terms of anthelmintic treatment programmes. Surveys in Ireland and in the Netherlands have shown the presence of eggs on the coat of owned dogs with a percentage of 8.8% [42] and 12.2% [40] respectively. However, eggs in both studies were not infective. Relatively old private dogs have been found with a higher percentage of eggs on their coats than puppies [37,40,42]. Additionally, the absence of a correlation between intestinal worm burden and intensity of coat contamination suggests that pick-up from a contaminate soil is the main reason for the presence of parasite eggs on the coat of a dog [6,40].

Dogs with patent toxocarosis do not represent an immediate risk for human infection for a variety of reasons [44-46] and direct contact with an infected dog is considered of minor importance in the zoonotic transmission of intestinal nematodes [47,48].

Canid ascarids can cause different syndromes (e.g. *visceral*, *neural* or *ocular larva migrans*, covert toxocarosis) in human beings, especially children and toddlers.

In fact, children are the subjects at highest risk of infection, due to exposure to areas (e.g. sandpits, green areas, gardens, playgrounds) potentially contaminated by *T. canis* eggs [44]. Children suffering by geophagic pica caused by mineral deficiency or behavior disorders are also at high risk [44,49]. For example, the impact of human infection by larval *Toxocara* in childhood is demonstrated by the hundreds of cases of blindness and eye damage calculated to occur yearly in the USA, which in the past has often led to eye enucleations due to misdiagnosis with retinoblastomas [3,48,50]. However, the role of migrating larvae of the feline ascarid *Toxocara cati* has been repeatedly also evocated in causing human syndromes [5]. Thus, the importance of environmental contamination by *T. cati* should not be neglected considering the likely absence of differences in terms of zoonotic potential between dog and cat roundworms [51]. People with a soil-related job (e.g. mechanics, gardeners, farmers, street cleaners) may be at more risk of infection with toxocarosis, as shown by their higher seroprevalence compared with values found in people with non-soil related occupations [52].

A survey from Ireland showed that garden soil contamination is not associated with the household presence of pets [53]. In general, ownership of companion animals is not definitively associated with seropositivity and seroprevalence for toxocarosis [52-54]. Contrariwise, human seropositivity to *Toxocara* spp. has been put in relation with the contamination of soil with parasite eggs in some US areas [55], although actual risk factors for human infections may change according to different geographical and epidemiological settings [56,57]. A study carried out in a city of Brazil showed that almost all seropositive children had the behavior disorder of geophagy and that they played nearly every day of the week in public squares with a minimum contamination of 1 *Toxocara* egg/gram of sand [58]. Additionally, it was also shown that contamination in the neighborhood of domiciles in the same areas was again positively correlated with seropositivity in children in the presence of infected animals. Interestingly, seronegative children played infrequently in public squares [58].

Zoonotic hookworms may cause different pictures of skin, enteric and pulmonary diseases, being the *cutaneous larva migrans* the most important. Interested readers are referred to [7,8,59]. A relationship between the presence of *Ancylostoma* spp. larvae in soil of public squares and occurrence of *cutaneous larva migrans* in children has been demonstrated in Brazil [60].

It is obvious that tourists sunbathing on beaches in risky areas where zoonotic hookworms are endemic are at risk of infection with larval hookworms.

The dog whipworm *T. vulpis* is not included in zoonotic intestinal nematodes of pets [48] and its zoonotic potential is questioned although presumed cases of *visceral larva migrans* and of patent intestinal infections have been described in people. At the moment *T. vulpis* cannot be ultimately considered as a zoonotic canine parasite and readers interested may find more details in [9].

Despite its high zoonotic potential, few references are available on the presence of *Strongyloides stercoralis* in public areas. For instance, *S. stercoralis*-like larvae have been found in soil samples from Iran [61] and Nigeria [62].

### Contamination and geography

Eggs of *Toxocara* spp., eggs and larvae of *Ancylostoma* spp. and eggs of *T. vulpis* have been found from soil and faecal samples in public areas from Europe, the Americas, Africa and Asia.

Table 1 reports key examples of surveys carried out in different countries to evaluate the frequency of canine parasites due to faecal pollution in various human settings.

**Table 1:** **Key examples of studies that evaluated the frequency** (%) **of soil contamination of public areas by roundworm**, **hookworm and whipworm eggs in different continents**

**Geographical area**	**Site**	**Frequency (%)**			**Reference**
		**Roundworms**	**Hookworms**	**Whipworms**	
**Africa**					
Niger	Kaduna		9.0		[63]
**Americas**					
USA	Connecticut	14.4			[64]
Argentina	Buenos Aires	13.2			[65]
	Buenos Aires	1.7	20.5	2.6	[66]
Brazil	Fernandopolis	79.4	6.9		[67]
	Itabuna		47.9		[68]
	São Paulo	29.7			[69]
	Guarulhos, São Paulo	68.1	64.8		[70]
Chile	Santiago	66.7			[71]
Venezuela	Ciudad Bolívar		61.1		[72]
**Asia**					
Japan	Tokushima	63.3			[73]
Thailand	Bangkok	5.7			[74]
Turkey	Ankara	45.0			[75]
**Europe**					
Ireland	Dublin	15.0			[76]
Spain	Madrid	16.4	3.0		[77]
Italy	Marche region	33.6			[78]
	Milan	7.0	3.0	5.0	[79]
	Bari	2.5	1.6	2.5	[80]
	Naples	0.7-1.4	2.4	10.1	[81]
	Messina	3.6	2.6	1.3	[82]
	Alghero	0.5-8.0	4.0	1.9	[83]
Poland	Wrocław	3.2	4.9	4.9	[84]
	Warsaw	26.1			[85]
	Kraków	15.6-19.8			[86]
Turkey	Erzurum	64.3			[87]
Czech Republic	Prague	20.4			[88]
Hungary	Eastern and northern areas	24.3-30.1	8.1-13.1	20.4-23.3	[89]
Slovak Republic	Bratislava	18.7			[90]

In a recent survey, canine faecal deposits were collected from June 2012 to January 2013 in public green areas (e.g., historic gardens, children’s playgrounds or green places for physical activities or fitness) in three different municipalities of Italy (i.e., Padua, Rome and Teramo). Out of a total of 677 collected samples, 38 (5.6%) scored positive upon copromicroscopical examination for at least one canine geo-helminth, i.e. 22/209 (10.6%) from Rome, 13/198 (6.6%) from Teramo, and 4/270 (1.5%) from Padua. Overall, the highest prevalence was detected for *T. vulpis* (30; 4.4%), followed by *T. canis* (13; 1.9%), and *A. caninum* (3; 0.4%), distinguished from *Uncinaria* based on the egg size differences reported in literature [91,92]. More specifically, prevalence values for *T. vulpis* and *T. canis* showed a similar trend in each municipality (7.7% and 1.9% in Rome, 5.1% and 3.6% in Teramo, 1.5% and 0.7% in Padua, respectively), whereas *A. caninum*-positive samples (1.4%) were observed solely in Rome (unpublished data).

Although parasite eggs may be found in several urban and industrialised settings, the risk of environmental contamination is particularly relevant in resource poor communities due to the fact that extensive worm control programs are limited by financial constraints. Also, in those poor settings the public health system is deficient, there is usually a high number of stray and feral animals and people lack awareness of health risks [93]. In these settings the bond between physicians, veterinarians and the whole community should be re-enforced to minimize as much as possible the risk of public hazard.

### What can we do to reduce environmental contamination?

A reduction of the contamination of public areas by dog helminths can be achieved only with a combination of approaches, e.g. reliable worm control programs, awareness of veterinarian and behavior of pet owners and the general public.

No reliable methods exist to realistically eliminate eggs or larvae of intestinal nematodes of pets present on the ground. Therefore, preventing the initial contamination of the environment is of paramount importance. The individualized treatment of parasitized animals is mandatory to control infection in pets and environmental pollution. Unfortunately, negligence in performing diagnostic copromicroscopy in veterinary practices is frequent, due to the fallacy in considering an antiparasitic treatment powerful enough to “generically clear parasites”.

Contrariwise, copromicroscopic examinations should be regular for pets, given that virtually all dogs are at risk of becoming infected by intestinal nematodes for all their life. The role of veterinarians is crucial, because pet owners should be convinced of the importance of periodic faecal examinations. Veterinarians have a plethora of parasiticides, which can be administered according to each individual possible *scenario* and both owner and animal compliance to treat infected animals [7,9]. Thorough indications for worm control programs have been released by the US Companion Animal Parasite Council (CAPC) and the European Scientific Counsel Companion Animal Parasites (ESCCAP) [7,94,95].

A key point for controlling pet parasites is the lifelong chemopreventative program. Using year-round treatment is of importance where there is the necessity to perform the annual chemoprophylaxis for other severe parasites and not only for intestinal nematodes, e.g. for the prevention of cardio-pulmonary nematodes, i.e. *Dirofilaria immitis* and *Angiostrongylus vasorum*. In addition, several formulations containing compounds effective against intestinal nematodes also contain cestocides which are powerful for controlling infections caused by tapeworms distributed worldwide (e.g. *Dipylidium caninum*) or hazardous for humans (e.g. *Echinococcus* spp.).

Broad-spectrum formulations with an easy mode of administration (e.g. chewy tablets, spot-on) fit particularly with year-long worm control programs. Faecal examinations should be performed whether or not a monthly-based treatment program is used, even when the dog appears healthy, as there are parasites that may not be covered by the treatment program or there may be poor compliance with the program. In fact, owners may be not interested in paying for faecal examination if the animals are asymptomatic, because they are commonly considered parasite-free. While puppies and their thousands of eggs shed daily are the major source of contamination for the environment, a US study has shown that after young dogs, the most parasitized category of pets are > 10 years old [96]. This high degree of parasitism in old animals could reside in a lack of willingness of owners in chemopreventative and/or worm control programs in old pets [96]. Indeed, there is no reason to consider an old animal a less effective source of infection for pets, human beings and the environment.

Unfortunately, public risk perception and awareness may be poor in veterinarians, the general public and pet owners of several countries [97-100]. Interview- based studies have been conducted to understand how the risk perception is present in the human population and to implement awareness of the general public and of pet owners. For example, a British survey has unveiled that less than the half of the participants (i.e. pet and non-pet owners) were aware of the potential for transmission of parasites *via* animal faeces with no differences between who had a pet and who did not [55].

Similarly, a recent Italian interview-based study carried out during summer 2012 in the cities of Rome and Padua illustrated that out of 469 participants, 246 (52.5%) were aware of the health risk associated with canine faecal pollution in urban settings, with no differences between pet and non-pet owners. In the same study, the awareness of the health risks was higher in Padua (205/339, corresponding to 60.4%) than in Rome (41/130; 31.5%), again with no differences between pet and non-pet owners (unpublished data).

Veterinarians should routinely inform clients about source of infections for both pets and humans and on reliable measures to prevent transmission to other animals and people. Regrettably, this is not a frequent behavior. As a key example, less than the half of interviewed veterinarians in a Canadian survey discussed the zoonotic risk of pet ownership with clients, while the remainder did this only in particular cases or not at all [100].

**Figure 2:**
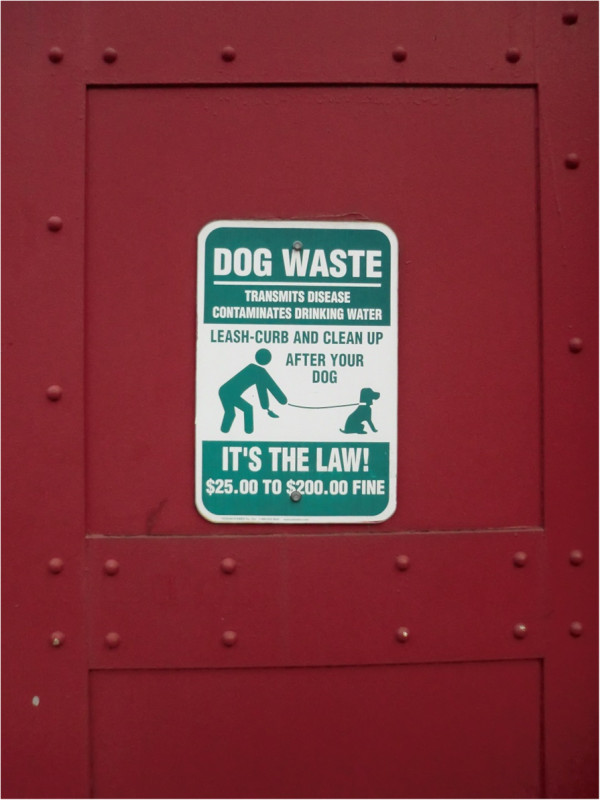
**Indication for dog-owners in New York City, USA.**

**Figure 3:**
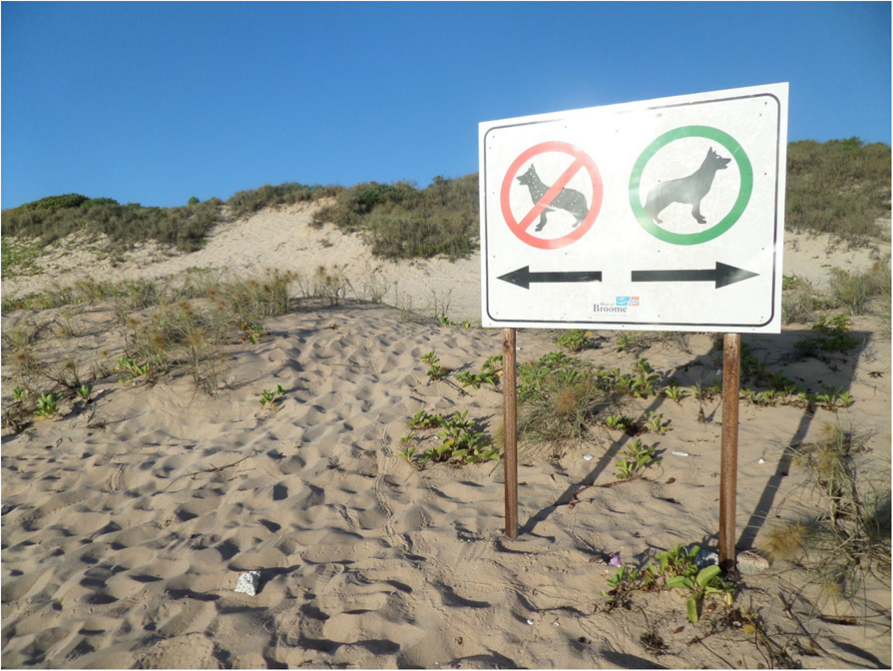
**Beach area reserved for dogs in Broome, Australia.**

Given that public squares, sandpits, playgrounds, beaches are always at a high risk for heavy contamination by pet faeces and public parks and green areas are always contaminated by parasites of dogs [4,8,101-103], avoiding animal defecation in public areas or immediate collection of stool by the pet owner is crucial (Figure 2). Veterinarians should educate owners on regular removal and disposal of faeces, which is at the basis to minimize environmental contamination and risk of transmission [44,48]. When walking their pets in public areas, all owners should respect local indications and keep their animals in reserved areas, if present (Figure 3).

A very “creative” measure was recently adopted by the Municipality of Brunete (Spain), in which undercover volunteers were recruited to patrol the streets, and to confront dog-owners who did not remove the faeces of their pet. Approaching the guilty owner for a friendly conversation, volunteers swindled some useful information to identify his/her domicile by the preexisting pet-registration database. At the end of the conversation, when the dog-owner was out of sight, the volunteers picked up the dog’s faeces, the excrements were boxed, and hand-delivered to the pet owner along with an official fine and warning [104].

Other than constant municipal cleaning and maintenance, controlled access of green areas and public parks by fences is an effective way of prevention of faecal contamination. A study in Japan has shown that placing vinyl plastic covers over sandboxes at night is able to discourage animals from defecating there [105]. An extreme measure chosen by some municipalities is the elimination of sandboxes from parks and playgrounds [8]. It is important to note, however, that while *T. canis* eggs are most prevalent in public parks, sandboxes are mainly contaminated with eggs of *T. cati* due to the common behavior of cats during defecation [103].

Surveillance of the presence of parasite eggs in public soil is also important in this integrated approach to control intestinal parasites. In general, microscopic examination of soil samples is performed to identify *Toxocara* eggs, although this method may have low sensitivity and specificity [106,107]. DNA-based approaches have been developed to discriminate eggs of ascarids in soil samples [107,108], although some pitfalls may impair a routine use, e.g. low throughput analysis and risk of carry-on contamination. A duplex Real-Time PCR has been recently validated for the detection and discrimination of *T. canis* and *T. cati* eggs in different samples, including soil. This assay is promising for the implementation of standardized methods able to evaluate the presence of roundworm eggs in contaminated soil on a large scale. In particular, this novel molecular tool can be used to investigate, with a high throughput, the occurrence and the level of contamination of eggs of *T. canis* (and *T. cati*) in urban parks, green areas, playgrounds and sandpits [109].

This is of importance because different investigations have shown that some urban environments may be heavily contaminated by *T. cati* rather than by *T. canis*[101].

## Conclusion

**Figure 4:**
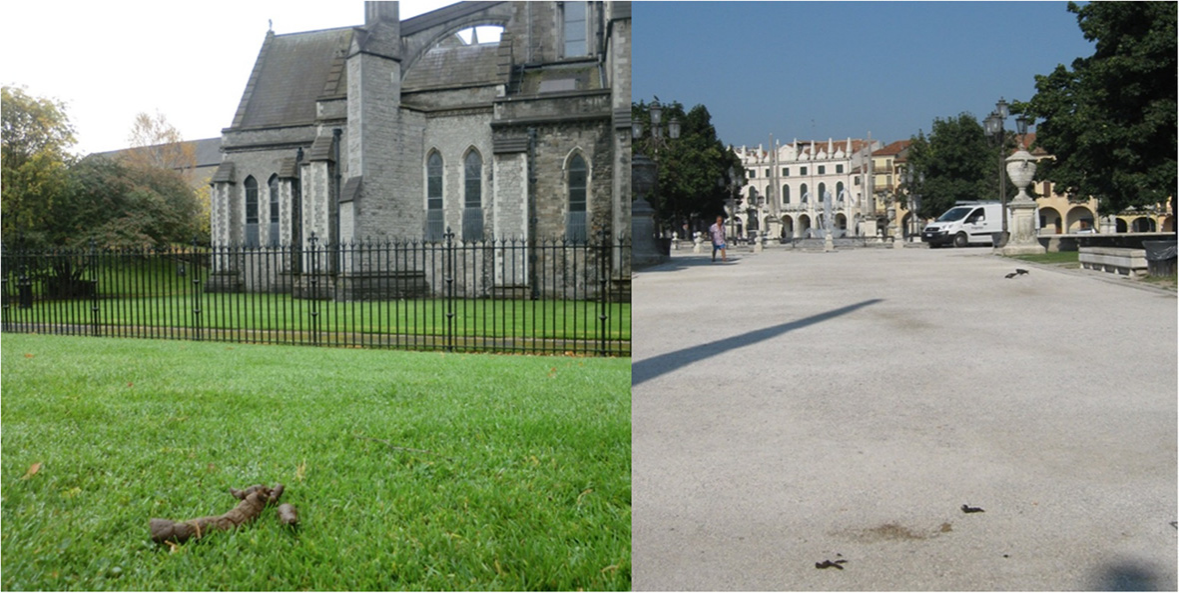
**Dog faeces in a green park of Dublin, Ireland (left) and in a public square of Padua, Italy (right).**

Canine faeces in cities are an important source of pathogens for the pet population, for dog owners and for the community in general. Prevention of initial contamination is the most important way to avoid human and animal infections, given that no practical methods are available to actually minimize environmental egg contamination. The non-polite habit of dog owners of not removing feces of their pet from streets and green areas (Figure 4) represents a concern for hygiene and health of both animals and humans. Hence, polluted public environments represent the principle risk for human health with zoonotic intestinal nematodes of dogs [38]. Other than social responsibility in eliminating dog faeces from streets, parks and squares, appropriate worm control programs, especially in young dogs, are crucial to control faecal contamination and minimize the risk of infection for humans and other animals.

Unfortunately, public education in reducing the risk of exposure for both humans and companion animals is poor. In recent years sociological changes have influenced the relationships between physicians and veterinarians, towards the concept of the “One Health Program” (i.e. “*the collaborative work of multiple disciplines to help attain optimal health of people*, *animals*, *and our environment*”) [110]. Thus, there is the necessity for physicians, veterinarians and the general public to foster interest and efforts in appropriate control programs towards a reduction of pollution of the cities and of the risk of infection for both animals and people.

## Competing interests

Studies whose unpublished results are reported in the review were financed by Novartis Animal Health, of which FLT and JD are employees.

## Authors’ contributions

DT, AFdR and MP conceived the article and all authors contributed to its drafting, preparation and intellectual content. AFdR and MP were scientifically responsible for the studies whose unpublished results are reported in the review. All authors read and approved the final manuscript.
